# The Work Productivity and Activity Impairment (WPAI) questionnaire

**DOI:** 10.1093/occmed/kqaf097

**Published:** 2025-09-14

**Authors:** Karen Walker-Bone

**Affiliations:** School of Public Health and Preventive Medicine, Monash Centre for Occupational and Environmental Health Research, Monash University, Melbourne, Victoria 3004, Australia

## A BRIEF HISTORY OF THE QUESTIONNAIRE

Developed for clinical trials by health outcomes researchers, the Work Productivity and Activity Impairment (WPAI) questionnaire was designed to enable quantitative assessment of the impact of disease, symptoms or health conditions on work (paid and unpaid) [1,2].

## DESCRIPTION OF THE QUESTIONNAIRE

The WPAI was designed to measure absenteeism, presenteeism and activity impairment attributable to ill-health over the preceding 7 days.

It is a 6-item questionnaire with the first question discriminating between participants who do paid work from those doing unpaid or no work (the latter groups are diverted to question 6 only). Amongst people currently doing paid work, questions 2–4 measure the following: absenteeism (‘how many hours did you miss from work because of your health problems?’), hours missed from work for non-health reasons, and hours actually worked (in each case, the questionnaire leaves a blank space followed by the word ‘hours’ so that responses are unrestricted). Self-assessed productivity is measured by question 5, which asks ‘how much did health problems affect your productivity while you were working?’. Responses are recorded on a visual analogue scale (VAS) (range 0–10), which is labelled at the zero end ‘health problems had no effect on my work’ and at the 10 end ‘health problems completely prevented me from working’. Question 6 is another VAS (also range 0–10) with the words ‘consider only how much health problems affected your ability to do your regular activities, other than work at a job’ and is completed by all participants not only those in paid work.

The WPAI calculates percentage impairment (maximum 100%) with higher numbers representing greater work impairment. Scores for each item are multiplied by 100 to calculate percentages. The results are presented as: percentage of work time missed due to health (incorporates responses to questions 2 and 4); percentage of impairment while working due to health (response to question 5); percentage of overall work impairment due to health (computed from responses to questions 2, 4 and 5); and percentage of activity impairment due to health (response to question 6). Coding rules (including how to deal with missing data) are available online (http://www.reillyassociates.net) [2].

## THE CONTEXTS IN WHICH THE QUESTIONNAIRE IS TYPICALLY USED

There are three versions of the questionnaire, one for general ­ill-health (the ‘General Health’ version: WPAI: GH), another for ‘Specific Health Problems’ (WPAI: SHP) and a combined version (WPAI: GH/SHP) which enables summation of work impairment from one specific health condition and all health problems [2]. The authors recommend that the General Health version is more suitable for use by people with systemic diseases or individuals with multimorbidity, who may find it difficult to attribute their impairments to one condition. The Specific Health Problems questionnaire is more suited to localised or specified conditions, e.g. hand dermatitis, asthma and irritable bowel syndrome.

## VALIDATION

Compared to many work outcome measures, the WPAI has become widely used and its performance and properties have been measured extensively. On their website, the WPAI authors provide a bibliography of studies validating the tool for a specific health condition, validating a translated version, or testing the psychometric properties of the tool [2]. Several hundred citations are listed on the website, but there are no citations since 2015. However, a search of PubMed revealed over 2000 total citations when searching ‘WPAI’ and over 1000 searching ‘validation & WPAI’, with many dated since 2015 indicating the measure continues to be widely used.

Despite its extensive validation and testing, it should be borne in mind that there are differences between measuring absence from work and effectively quantifying productivity. Absence from work is relatively straightforward to quantify over the past seven days without much risk of recall bias. However, assessment of productivity loss relies solely on a subjective rating by the worker. In some types of work, productivity may be relatively easy to quantify, e.g. manufacturing, but when health affects the quality of work, it can be far more difficult to accurately quantify lost productivity. Importantly for safety critical roles, poor quality work due to ill-health could cause serious injury or fatality. A study aiming to compare four different self-reported measures of lost productivity, one of which was the WPAI, with employer metrics of productivity found that productivity loss questionnaires correlated only weakly/moderately with employer economic metrics and also correlated poorly with each other [3]. Thus, although the WPAI is well-validated and widely used, more objective measures of lost productivity are also urgently needed, either to use alongside WPAI or even replace it.

## KEY RESEARCH USING THE WPAI

The WPAI has been widely used to study a diverse range of symptoms (e.g. pain, sleep, dyspepsia, headache, overactive bladder, erectile dysfunction, headache), specific conditions (e.g. cancer, anaemia, mental health, asthma/allergies, hepatitis, inflammatory bowel diseases, mental health, diabetes, COPD), amongst patients using primary or secondary healthcare services and in clinical trials [2]. It has been used to assess the impact of health conditions or symptoms on an individual’s work performance, as well as longitudinally to measure the effectiveness of workplace interventions on worker health [4,5]. The WPAI has also been used for assessment of the impact of health conditions on caregivers/family members and among specific occupational groups, e.g. ‘fly-in, fly-out’ workers in mining [6], shift workers [7] and nurses [8].

Musculoskeletal researchers have increasingly acknowledged the importance of measuring work participation as a core outcome for their intervention studies [9]. An Outcome Measures in Rheumatology (OMERACT) group has led projects for over 10 years [10,11] including evaluating five different measures of work productivity with 70 patients with inflammatory arthritis or osteoarthritis drawn from seven European countries [12]. Among the measures considered, patients reported that the 7-day recall period used in the WPAI tool was appropriate and, of all measures, it was considered the most relevant to them (by 29% of patients). Additionally, the OMERACT group assessed construct validity of the WPAI and some psychometric properties, finding it performed moderately well [13]. Since then, the WPAI has been increasingly selected as the productivity measure for use in audits, trials and registries by musculoskeletal researchers, including the national UK Health Quality Improvement Partnership early inflammatory arthritis audit [14].

## AVAILABILITY

The questionnaire is readily available online [2] at no cost and can be used without the requirement for written permission or licence. It has been translated into at least 140 languages.

## Data Availability

No original data were generated for this review.
